# Wound-induced polyploidization is dependent on Integrin-Yki signaling

**DOI:** 10.1242/bio.055996

**Published:** 2021-01-25

**Authors:** Rose Besen-McNally, Kayla J. Gjelsvik, Vicki P. Losick

**Affiliations:** 1 Biology Department, Boston College, Chestnut Hill, MA, 02467, USA; 2 Graduate School of Biomedical Sciences and Engineering, University of Maine, Orono, ME, 0×4469, USA; 3 Kathryn W. Davis Center for Regenerative Biology and Aging, MDI Biological Laboratory, Bar Harbor, ME, 04609, USA

**Keywords:** *Drosophila*, Focal adhesion kinase, Integrin, Polyploidy, Wound healing, Yorkie

## Abstract

A key step in tissue repair is to replace lost or damaged cells. This occurs via two strategies: restoring cell number through proliferation or increasing cell size through polyploidization. Studies in *Drosophila* and vertebrates have demonstrated that polyploid cells arise in adult tissues, at least in part, to promote tissue repair and restore tissue mass. However, the signals that cause polyploid cells to form in response to injury remain poorly understood. In the adult *Drosophila* epithelium, wound-induced polyploid cells are generated by both cell fusion and endoreplication, resulting in a giant polyploid syncytium. Here, we identify the integrin focal adhesion complex as an activator of wound-induced polyploidization. Both integrin and focal adhesion kinase are upregulated in the wound-induced polyploid cells and are required for Yorkie-induced endoreplication and cell fusion. As a result, wound healing is perturbed when focal adhesion genes are knocked down. These findings show that conserved focal adhesion signaling is required to initiate wound-induced polyploid cell growth.

## Abstract (precis)

**Summary:** Conserved focal adhesion genes, mys and Fak, enable efficient wound repair by inducing polyploid cell growth via cell fusion and Yki-dependent endoreplication.

## INTRODUCTION

Tissue repair requires either the proliferation or growth of cells to compensate for cell loss. Cells can grow in size by becoming polyploid, as cell size scales with DNA content. Many invertebrate and vertebrate organs depend on polyploid cell growth for tissue repair and regeneration (Gjelsvik et al., 2019; Lazzeri et al., 2019), including the mouse hepatocytes in the liver and tubule epithelial cells in the kidney as well as the zebrafish epicardium in the heart (Cao et al., 2017; Lazzeri et al., 2018; Wilkinson et al., 2018; Zhang et al., 2018). *Drosophila* tissues also induce polyploid cell growth in response to tissue damage in the abdominal epithelium, follicular epithelium, pyloric hindgut, and intestinal epithelium (Cohen et al., 2018; Losick et al., 2013; Tamori and Deng, 2013; Xiang et al., 2017). Despite many examples of polyploidy in tissue repair and regeneration, the signals required to initiate polyploid cell growth in response to injury remain poorly understood.

Wound-induced polyploidization (WIP) occurs in the *Drosophila* abdominal epithelium, where a giant polyploid cell forms by both endoreplication and cell fusion (Losick, et al., 2013). The endocycle compensates for cell loss by precisely restoring epithelial synthetic capacity, whereas cell fusion speeds wound closure (Losick et al., 2013, 2016). These studies also revealed that endoreplication was dependent on the conserved Hippo-Yorkie (Yki) signal transduction pathway, which has been found to control the cell cycle and growth (Oh and Irvine, 2010). In WIP, Yki transcriptionally induces expression of *Myc*, *E2F1*, and *cycE*, which are required and sufficient for endoreplication in this model (Grendler et al., 2019). In mammals, YAP, the ortholog of Yki, was also shown to regulate endoreplication, but via acetylation of the cell cycle inhibitor Skp2, resulting in mitotic arrest and tumorigenesis of hepatocytes in the mouse liver (Zhang et al., 2017).

The Hippo pathway regulates Yki/Yap activation by responding to biological and biophysical cues, including adhesion, polarity, extracellular matrix (ECM) stiffness, and cytoskeleton rearrangement (Pocaterra et al., 2020; Zheng and Pan, 2019). Cell-ECM adhesion is mediated by integrin and the focal adhesion complex. In mammals, Hippo-Yap signaling was found to be dependent on the Enigma protein family and focal adhesion kinase, which signals to Hippo via the PI3K pathway (Elbediwy et al., 2016; Kim and Gumbiner, 2015). However, both of these studies were performed in cell culture and it remains unknown whether similar signaling pathways dictate polyploid cell growth *in vivo*. Here, we find that conserved focal adhesion proteins, including integrin and focal adhesion kinase, are upregulated in wound-induced polyploid cells and are required to activate Yki to induce WIP.

## RESULTS AND DISCUSSION

### Focal adhesion proteins are induced and required for endoreplication during WIP

**Fig. 1.**
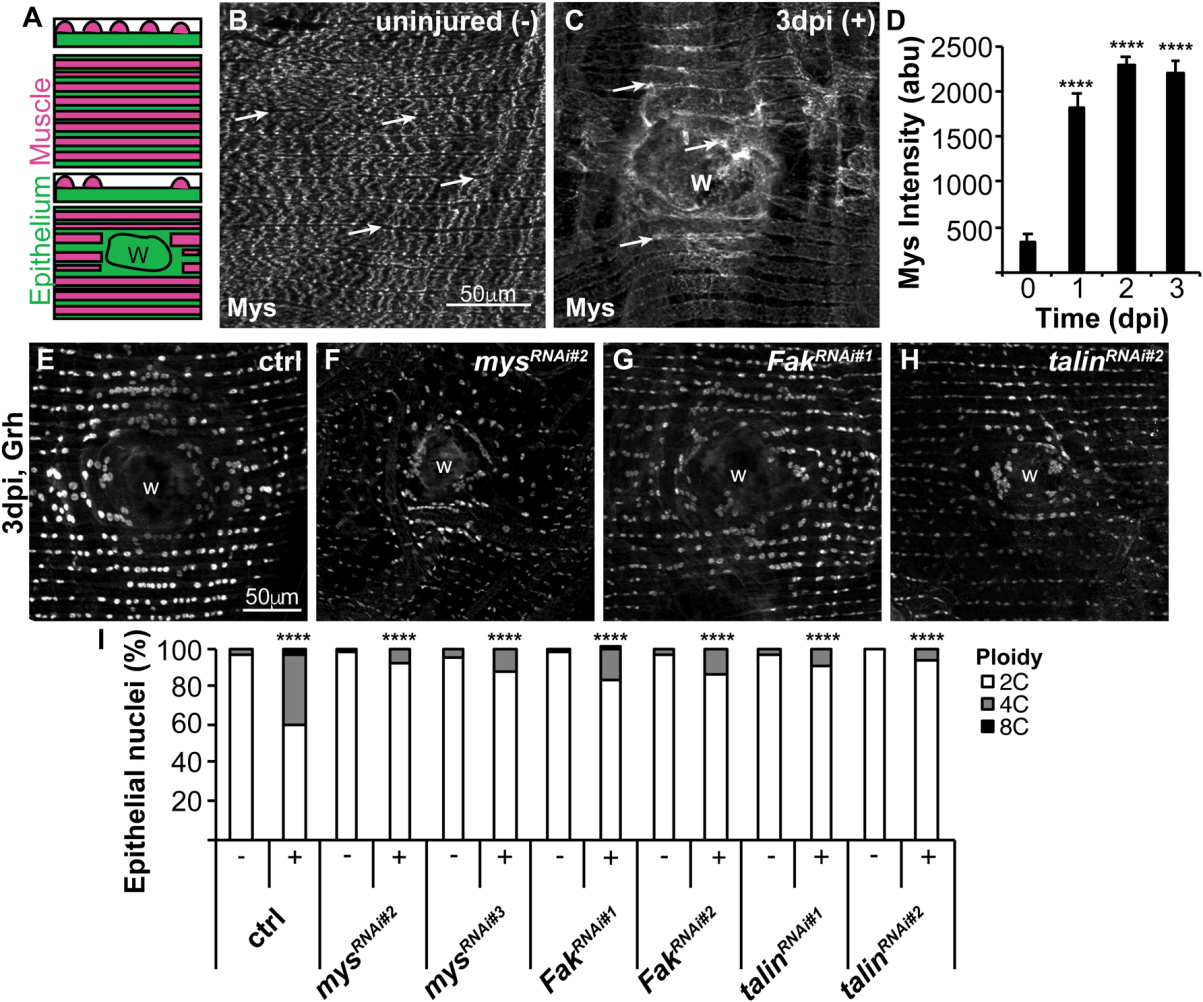
**Focal adhesion genes are induced and required for endoreplication.** (A) Illustration of the adult *Drosophila* abdominal organization of the lateral muscle fibers (red), overlaying the epithelium (green) in the transverse, z-view (top) and flattened, x-y view (bottom). Epithelial gene expression can be observed and measured in the gaps between overlaying muscle fibers. After injury the epithelium, but not the muscle fibers are repaired over the wound scar (outlined, w). (B) Representative immunofluorescent images of mys staining in the (B) uninjured (−) and (C) 3 dpi (+) adult fly abdomen. Epithelial mys expression is marked by arrows. Wound site, w. (D) Time course of *mys* expression quantified in the epithelium at 0 dpi (*n*=5), 1 dpi (*n*=12), 2 dpi (*n*=13), and 3 dpi (*n*=12). Error bars represent mean±s.e. and data were analyzed by two-tailed unpaired *t*-test. (E–H) Representative immunofluorescent images of control, *mys^RNAi^*, *Fak^RNAi^*, and *talin^RNAi^* at 3 dpi stained with epithelial nuclear marker (Grh). (I) Quantification of epithelial ploidy in the control (−, *n*=15 and +, *n*=12), *mys^RNAi#2^* (−, *n*=12 and +, *n*=10), *mys^RNAi#3^* (−, *n*=11 and +, *n*=9), *Fak^RNAi#1^* (−, *n*=12 and +, *n*=8), *Fak^RNAi#2^* (−, *n*=7 and +, *n*=7), *talin^RNAi#1^* (−, *n*=5 and +, *n*=4), and *talin^RNAi#2^* (−, *n*=4 and +, *n*=3). Data were analyzed by two-way ANOVA with Tukey's multiple comparisons test. Also see Fig. S1 and Table S1.

A needle puncture wound through the *Drosophila* abdomen triggers WIP (Losick et al., 2013). First, at 1 h post injury, a melanin scab forms sealing the damaged cuticle. Then, the epithelium repairs by 3 days post injury (dpi) through generation of multinucleated, polyploid cells by endoreplication and cell fusion. The ventral fly epithelium is overlaid by lateral muscle fibers, which remain unrepaired and permanently severed (Fig. 1A). We previously found that Hippo-Yki signaling was required for WIP, initiated at the site of wound scab, where permanent ECM remodeling occurs (Losick et al., 2013, 2016). ECM remodeling has been shown to signal via focal adhesion proteins, integrin, talin, and Fak (focal adhesion kinase) to regulate Hippo-Yap signaling in mammalian cell culture models making the focal adhesion complex a candidate WIP activator (Elbediwy et al., 2016; Kim and Gumbiner, 2015).

We first examined the expression and localization of three conserved focal adhesion proteins in *Drosophila*: integrin [myospheroid (mys)], Fak, and talin. We found that Mys is strongly expressed in the lateral muscle fibers that overlay the abdominal epithelium prior to injury and expressed at a low level in the underlying epithelium, as measured in gaps between muscle fibers (Fig. 1B, arrows). Mys then becomes significantly upregulated (7-fold) in epithelium during the wound healing time course, 1–3 dpi (Fig. 1C,D). This is more easily observed as the overlaying muscle fibers are severed by the injury and not repaired (Losick et al., 2013). The talin antibody staining was either not effective in adult fly epithelium or expression was too low to be detected. However, we were able to detect Fak, which was also upregulated (2-fold) in epithelium in response to injury (Fig. S1A,B,E).

Next, we used the Gal4/UAS-RNAi system to knockdown focal adhesion genes in the fly epithelium and determine their role in WIP. First, the knockdown efficiency was confirmed for both *mys* and *Fak* by comparing expression in control (no RNAi) with two UAS-mys^RNAi^ and UAS-Fak^RNAi^ lines expressed with the epithelial specific Gal4 (epi-Gal4) driver (Losick et al., 2013) (Fig. S1A–H). We assayed for endocycle entry using the thymidine analog EdU as an S phase marker, as cells undergo successive S phases with each endocycle during WIP (Bailey et al., 2020; Losick et al., 2016). At 2 dpi, we observed 178 EdU^+^ nuclei on average for the control around the wound site as previously reported, whereas *mys* knockdown reduced the mean number of EdU^+^ epithelial nuclei to 74 (*mys^RNAi#2^*) and 40 (*mys^RNAi#3^*) (Fig. S1I–K).

To confirm that epithelial ploidy was reduced, we directly measured nuclear ploidy in uninjured (−) and 3 dpi (+) epithelia. Our previous studies have shown that the uninjured epithelial nuclei are diploid (2C) and therefore can be used as an internal control to measure changes in the fly epithelial cell ploidy (Bailey et al., 2020; Losick et al., 2013). As expected at 3 dpi, we observed that 41% of epithelial nuclei were polyploid compared to only 4% in uninjured control epithelial cells (Fig. 1E,I). Epithelial-specific knockdown of *mys* resulted in significant reduction in polyploid nuclei at 3 dpi to 8% and 12% for *mys^RNAi#2^* and *mys^RNAi#3^* strains, respectively (Fig. 1F,I). We also found that knockdown of *Fak* and *talin* significantly reduced epithelial nuclear ploidy to 17% (*Fak^RNAi#1^*), 14% (*Fak^RNAi#2^*), 10% (*talin^RNAi#1^*), and 7% (*talin^RNAi#2^*) polyploid at 3 dpi (Fig. 1G–I). Therefore, conserved focal adhesion genes are required to induce efficient endoreplication post injury in *Drosophila*.

### Yki dependent gene expression requires mys and Fak

**Fig. 2.**
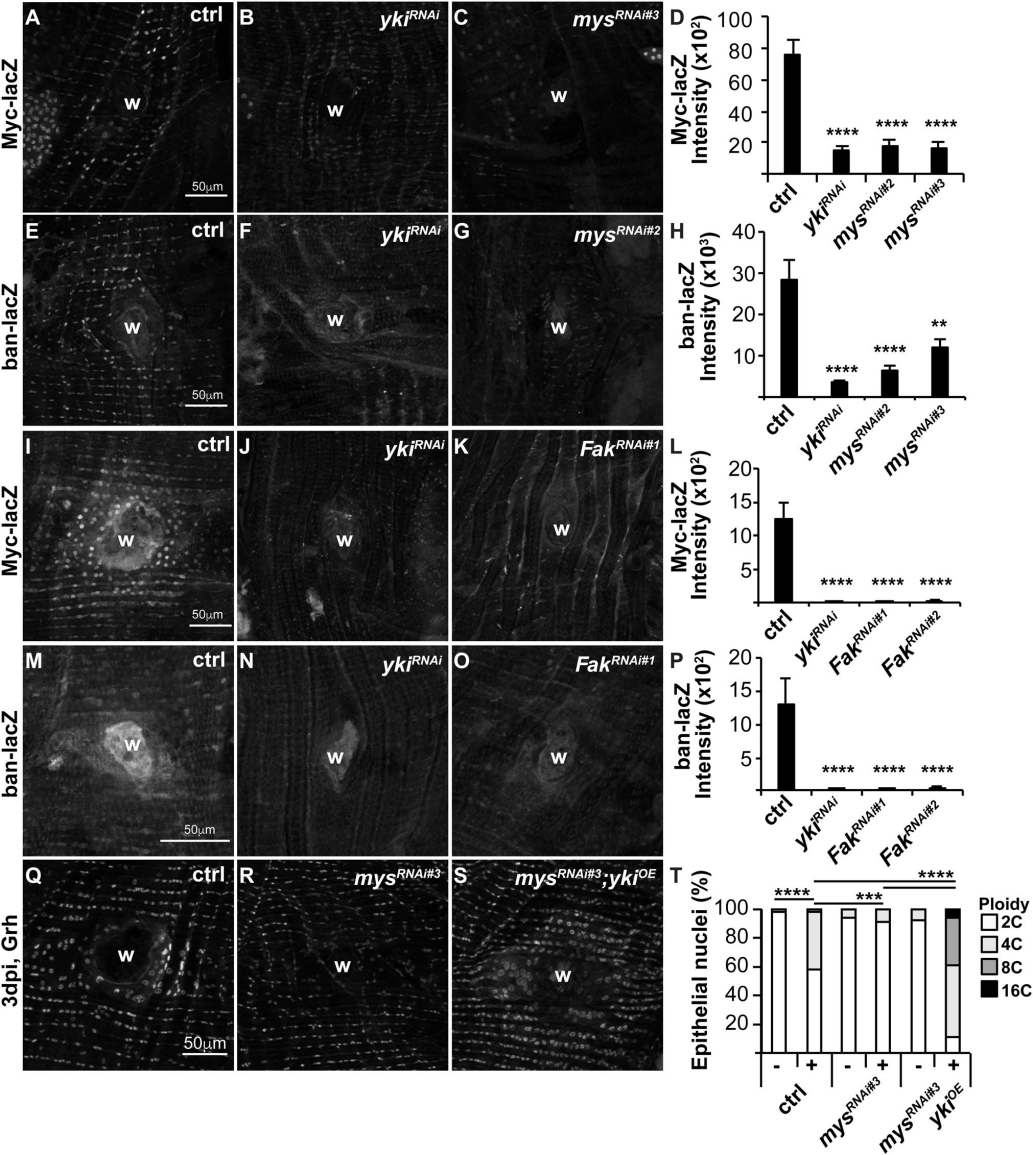
**Mys and Fak signal to Yki to regulate endoreplication.** Representative immunofluorescent images show expression of Yki-dependent reporters, Myc-lacZ and ban-lacZ at 2 dpi in control (A,E,I,M), *yki^RNAi^* (B,F,J,N), *mys^RNAi^* (C,G), and *Fak^RNAi^* (K,O). Wound site (w). Quantification of Yki reporters as shown Myc-lacZ (D, *n*=12, 12, 12, 12) and (L, *n*=9, 5, 9, 9) and ban-lacZ (H, *n*=12, 15, 11, 6) and (P, *n*=9, 5, 7, 8). Error bars represent mean±s.e. and data were analyzed by two-tailed unpaired *t*-test. (Q–S) Representative immunofluorescent images of control, *mys^RNAi^*, and *mys^RNAi^*; *yki^OE^* at 3 dpi stained with epithelial nuclear marker (Grh). (T) Quantification of epithelial ploidy in the control (−, *n*=10 and +, *n*=4), *mys^RNAi^* (−, *n*=8 and +, *n*=8), and *mys^RNAi^*; *yki*^OE^ (−, *n*=4 and +, *n*=3). Data were analyzed by two-way ANOVA with Tukey's multiple comparisons test. Also see Table S2.

We previously showed that Yki-dependent gene expression was required for endocycle entry post injury (Grendler et al., 2019). To test whether the focal adhesion complex is upstream of Yki activation, we assayed for expression of two known Yki targets, *Myc* and *bantam* (*ban*) whose expression can be detected with the lacZ reporters, Myc-lacZ and ban-lacZ, respectively*.* We found that knockdown of mys resulted in ∼4-fold reduction in Myc-lacZ and up to 4-fold reduction in ban-lacZ expression comparable to *yki^RNAi^* at 2 dpi (Fig. 2A–H). This suggests that mys is required to activate Yki post injury. Similarly, *Fak^RNAi^* resulted in a significant reduction of Myc-lacZ and ban-lacZ expression comparable to *yki^RNAi^* at 2 dpi (Fig. 2I–P). Therefore, focal adhesion signaling via mys and FAK are required to induce Yki dependent targets post injury.

Next, we asked if Yki overexpression is sufficient to rescue endoreplication when *mys* was knocked down. To test this, we generated an epi-Gal4/ UAS-mys^RNAi#3^; UAS-yki^OE^ fly strain and measured ploidy in uninjured (−) or 3 dpi (+) epithelia in comparison to control (epi-Gal4/ w^1118^) and *mys^RNAi#3^* alone. We previously observed that Yki overexpression induces hyper-polyploidization post injury (Losick et al., 2016). Here, we found that Yki restores endocycling when *mys* is simultaneously knocked down (Fig. 2Q–T). This further suggests mys acts upstream of Yki to induce endoreplication during wound repair.

### Mys and Fak are required for cell fusion and re-epithelialization

**Fig. 3.**
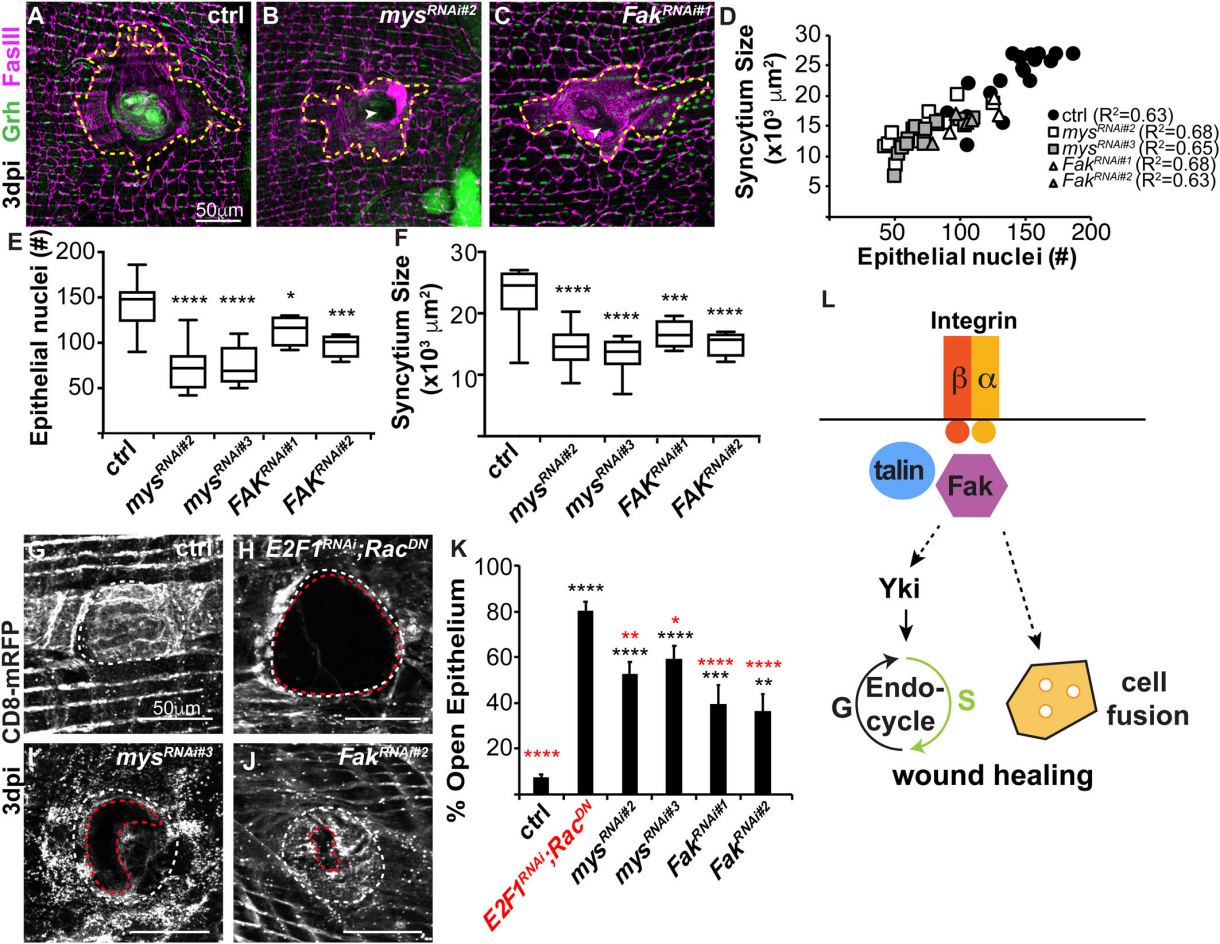
**Cell fusion and wound healing are dependent on mys and Fak.** (A–C) Representative immunofluorescent images of fly epithelium at 3 dpi. Epithelial nuclei (Grh, green), septate junctions (FasIII, magenta), giant syncytium (dashed yellow line) and wound site (w). (D) Quantification of epithelial syncytium size and number of epithelial nuclei at 3 dpi. (E) Number of epithelial nuclei and (F) syncytium size are significantly reduced at 3 dpi. (G–J) Re-epithelization during wound repair is detected by expression of a membrane-linked RFP under epi-Gal4 control. Representative immunofluorescent images for (G) control (epi-Gal4/+), (H) *E2F1^RNAi^; Rac^DN^*, (I) *mys^RNAi^*, and (J) *Fak^RNAi^* at 3 dpi. Outlined are wound scar (dashed white line) and open epithelial area (dashed red line). (K) Percent open area (open epithelial area/ wound scar size) at 3 dpi for control (*n*=34), *E2F1^RNAi^, Rac^DN^* (*n*=26), *mys^RNAi#2^* (*n*=17), *mys^RNAi#3^* (*n*=19), *Fak^RNAi#1^* (*n*=14), and *Fak^RNAi#2^* (*n*=14). Data were analyzed by two-way ANOVA with Tukey's multiple comparisons test. Comparisons to control (black) and *E2F1^RNAi^, Rac^DN^* (red). Also see Table S3. (L) Model illustrating how integrin (mys, β-integrin), talin, and Fak, initiate endoreplication and/ or cell fusion to promote polyploid cell growth during wound healing.

Another key process for WIP is cell fusion, which enables formation of the giant multinucleated cells required to reseal the epithelium under the wound scab (Fig. 3A,D). We investigated the effect of *mys* and *Fak* knockdown on cell fusion at 3 dpi and found that *mys^RNAi^* and *Fak^RNAi^* reduced syncytia sizes at the wound site compared to the control epithelium (Fig. 3A–D). At 3 dpi, the central syncytia in control flies are on average 22,952 µm^2^ with 141 epithelial nuclei, whereas knockdown of *mys* and *Fak* significantly reduced syncytium size to 13,940 µm^2^ with 74 nuclei and 15,864 µm^2^ with 106 nuclei, respectively (Fig. 3E,F). The syncytium size was still proportional to the number of epithelial nuclei (R^2^=0.63–0.68), even though the focal adhesion gene knockdowns significantly reduced cell fusion (Fig. 3D). This reduction in syncytium size was also not due to a change in wound size, as we found the melanin scar sizes were not statistically different in any of the fly strains (data not shown). Studies in the larval epidermis found that focal adhesion genes, including *mys*, are required to prevent ectopic cell fusion during homeostasis (Wang et al., 2015). However, in adult fly epithelium we find focal adhesions genes are dispensable and the uninjured epithelium is able to maintain its normal cellular junctions during homeostasis (Fig. S2). There were no significant changes in nuclear number or cell size with genetic loss of mys or Fak (Fig. S2E,F). Instead, *mys* and *Fak* are required post injury in the adult fly epithelium for optimal cell fusion during wound repair.

Previous studies have shown that wound closure is dependent on endoreplication and cell fusion, as these mechanisms act in conjunction to generate the polyploid cells required for wound closure (Losick et al., 2013). Integrin mediated focal adhesion plays a conserved role in wound closure, as it is essential for cell migration in a variety of tissues (Plotnikov and Waterman, 2013). We observed that genetic loss of *mys* and *Fak* resulted in breaches in FASIII labeled cell-cell junctions in the epithelial sheet overlaying the wound scar, suggesting a role for integrin in this wound closure model as well (Fig. 3B,C, arrowheads). We expressed a membrane-bound red fluorescent protein (UAS-CD8.mRFP) using epi-Gal4 and assessed the extent of re-epithelialization by measuring the percent of open epithelial area versus the wound scar size. We found that there was a continuous epithelial sheet in the control fly strain (epi-Gal4, UAS-CD8.mRFP/w^1118^) with less than 7% of epithelial area open at 3 dpi, whereas a WIP mutant (*E2F1^RNAi^*; *Rac^DN^*), which fails to heal due to inhibition of both mechanisms of WIP has 80% of the epithelial area open at the wound site as previously reported (Fig. 3G,H,K) (Losick et al., 2013). *Mys* and *Fak* knockdown caused intermediate defects in re-epithelization at 3 dpi and were not significantly different from each other (Fig. 3G–K). We found that *mys^RNAi^* caused between 52–59% of the epithelium to remain open, whereas *Fak^RNAi^* resulted in 36–39% of epithelial area to remain open (Fig. 3G–K). Next, we examined wounded flies 1 day later to see whether re-epithelialization defects caused a delay or block in wound closure. At 4 dpi, the *mys^RNAi^* re-epithelialization defect was reduced to 19–24% of epithelium open, suggesting that genetic loss of integrin causes a delay in wound closure (Fig. S3), unlike the WIP mutant (*E2F1^RNAi^; Rac^DN^*), which remains permanently open. The *mys^RNAi^* delayed wound closure may be due to the reduced, but not permanent block in cell fusion. We previously showed that endoreplication or cell fusion alone is sufficient for epithelial wound closure, but inhibition of both simultaneously inhibits wound healing in adult fly epithelium (Losick et al., 2013). Here knockdown of *mys* inhibits endoreplication, but only reduces cell fusion hence why there is likely a delay in wound closure. We also suspect there are other signals besides via focal adhesion complex that regulate cell fusion and formation of syncytium that remain to be identified. Taken together, we have found that conserved focal adhesion genes, *mys* and *Fak*, enable efficient wound repair by inducing WIP through cell fusion and Yki-dependent endoreplication (Fig. 3L).

## MATERIALS AND METHODS

### *Drosophila* husbandry and strains

The *Drosophila melanogaster* strains used were raised on corn syrup, soy flour-based fly food (Archon Scientific) at 25°C. *Drosophila* strains for this study were from Bloomington Stock Center (b), and VDRC (v) stock numbers are noted. GMR51F10-GAL4 (b38793) called epi-Gal4 (Losick et al., 2013), w^1118^ (b3605), UAS-yki^RNAi^ (v104523), UAS-mys^RNAi#2^ (b33642), UAS-mys^RNAi#3^ (v29619), UAS-Fak^RNAi#1^ (b33617), UAS-Fak^RNAi#2^ (b44075), UAS-talin^RNAi#1^ (b28950), UAS-talin^RNAi#2^ (b32999), UAS-E2F1^RNAi^ (v108837); UAS-RacDN (b6292) (Losick et al., 2013), UAS-yki^OE^ (Huang et al. 2005), Myc-lacZ (b12247); 51F10-Gal4 (Grendler et al., 2019), ban-lacZ (b10154), 51F10-Gal4, and 51F10-Gal4, UAS-CD8.RFP (Losick et al., 2013), and UAS-mys^RNAi#3^; UAS-yki^OE^ (generated in this study).

### Injury, dissection, and immunostaining

Adult female flies were injured, dissected, fixed and stained as recently reported (Bailey et al., 2020). Tissues were stained with antibodies (manufacturer and dilutions) as follows: mouse anti-FasIII (DSHB 7G10, 1:50), chicken anti-βgal (Abcam ab9361, pre-absorbed, 1:1000), rabbit anti-RFP (MBL PM005, 1:2000), rabbit anti-Grh (1:300) (Losick et al., 2016), and rabbit anti-Phospho-Fak (Tyr397) (Thermo Fisher Scientific 44-624G, 1:100). Secondary antibodies from Thermo Fisher Scientific were used at 1:1000 dilution and included: donkey anti-rabbit 488 (A21206), goat anti-mouse 568 (A11031) and goat anti-chicken 488 (A11039).

### Imaging and quantification of Mys, Fak, and Yki reporter expression

Tissue samples were imaged on a Zeiss Axiovert with ApoTome using a 40x dry objective and analyzed with Fiji imaging software (NIH). Full Z-stack images taken at 0.5 µm per slice and flattened into SUM of stacks projections for all analysis. Fluorescent intensities were measured for anti-Mys, anti-Fak, and anti-βgal in at least three representative areas of the fly epithelium. The integrated density per area was measured between the overlaying muscle fibers in uninjured flies and around the wound site where the muscle fibers had retracted in the injured flies. The integrated density was then divided by the area of the measurement to normalize the staining intensity. The Yki reporters, ban-lacZ and Myc-lacZ, are expressed in epithelial nuclei and were measured by creating an ROI map of at least 30 Grh^+^ epithelial nuclei around the wound site. These nuclear areas were then transferred to the corresponding β−gal SUM of stacks images, and integrated density minus the background staining was quantified.

### Ploidy assay and quantification

*Drosophila* epithelial nuclear ploidy was performed as recently reported (Bailey et al., 2020). All samples were imaged under the same conditions and settings with a 300 µm×300 µm area used for analysis. The normalized ploidy values were binned into the indicated groups: 2C (0.6–2.9C), 4C (3.0–5.9C), 8C (6.0–12.9C), and 16C (>12.9).

### Epithelial syncytium size and re-epithelialization assay

Cell fusion was quantified by outlining the FasIII cell-cell junctions of central syncytia and counting the number of Grh^+^ epithelial nuclei encompassed within the outlined area. In the uninjured epithelia, a 150×150 µm square was analyzed for multinucleated cells. Wound closure was measured by assessing the continuity of the epithelial sheet over the wound scar. *Drosophila* expressing a membrane-bound UAS-mCD8-RFP under epi-Gal4 control were scored by measuring both wound scar and the area of the unhealed (open) epithelium providing the percent open area, area=open epithelial area/ wound scar size.

### Replicates and statistical analysis

All experiments were performed in duplicate with at least three biological fly replicates measured and analyzed per condition. Statistical analysis was performed as indicated using unpaired *t*-test (Excel software) or ANOVA (Prism). Statistical significance indicated as follows: *P<0.05, **P<0.01, ***P<0.001, and ****P<0.0001.

## Supplementary Material

Supplementary information
