# Supplementary material for: Wound-induced polyploidization is dependent on Integrin-Yki signaling
Source: Biol Open. 2021 Jan 25;10(1):bio055996. doi: 10.1242/bio.055996 (PMC7860123; doi:10.1242/bio.055996)
Supplement: Supplementary information [file biolopen-10-055996-s1.pdf]

Table S1. Raw data and analysis from graphs in Fig. 1D and 1I.

Click here to Download Table S1

Table S2. Raw data and analysis from graphs in Fig. 2D, 2H, 2L, 2P, and 2T.

Click here to Download Table S2

Table S3. Raw data and analysis from graphs in Fig. 3D, 3E, 3F, and 3K.

Click here to Download Table S3

Table S4. Raw data and analysis from graphs in Fig. S1E, S1H, and S1K.

Click here to Download Table S4

Table S5. Raw data and analysis from graph in Fig. S2D-F.

Click here to Download Table S5

Table S6. Raw data and analysis from graph in Fig. S3D.

Click here to Download Table S6

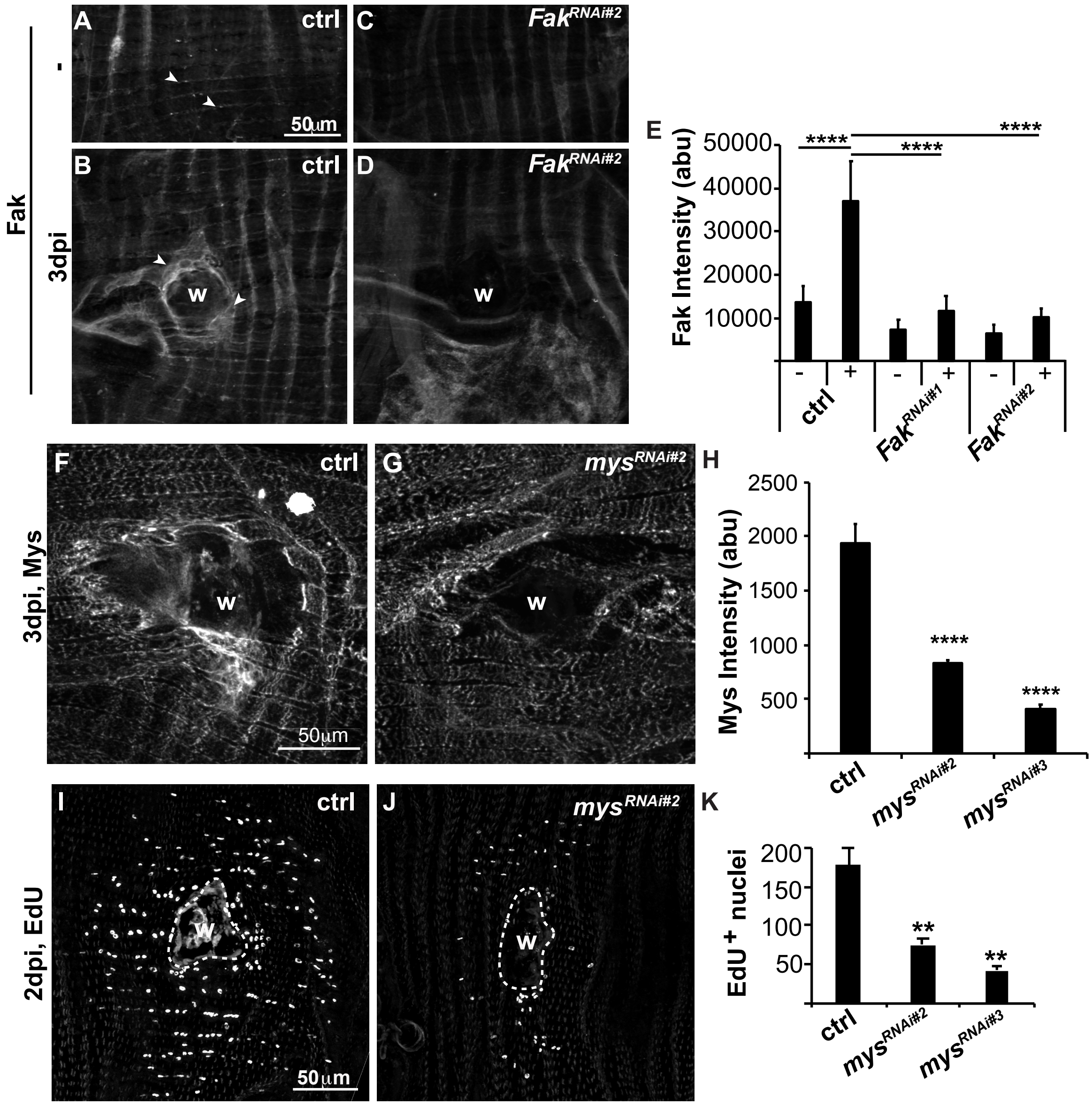

Figure S1. Fak expression, RNAi verification, and mys dependent endoreplication.

(A-D) Representative immunofluorescent images of Fak staining in the fly abdomen of the control (epi-Gal4/ w^{1118}) or Fak knockdown (epi-Gal4/UAS-$Fak^{RNAi\#2}$). Uninjured (-) and 3 dpi (+). Examples of epithelial Fak staining (arrowheads). Wound site (w). (E) Quantification of Fak epithelial expression in control (-, n=15 and +, n=11), $Fak^{RNAi\#1}$ (-, n=12 and +, n=7), and $Fak^{RNAi\#2}$ (-, n=9 and +, n=5). Error bars represent mean $\pm$ SE and data were analyzed by two-tailed unpaired t-test. (F and G) Representative immunofluorescent images of Mys staining in fly abdomen at 3 dpi in control (epi-Gal/w^{1118}) and $mys^{RNAi\#2}$ (epi-Gal/UAS-$mys^{RNAi\#2}$) strains. Wound site (w). (H) Quantification of Mys intensity in control (n=13), $mys^{RNAi\#2}$ (n=12), and $mys^{RNAi\#3}$ (n=5). Error bars represent mean $\pm$ SE and data were analyzed by two-tailed unpaired t-test, $p< 0.001$ (***). (I and J) Representative immunofluorescent images of EdU labeling (S phase cell cycle marker) at 2 dpi. Wound scar (outlined, w). (K) Quantification of EdU^{+} epithelial nuclei in control (n= 9), $mys^{RNAi\#2}$ (n=9), $mys^{RNAi\#3}$ (n=9). Error bars represent mean $\pm$ SE and data were analyzed by two-tailed unpaired t-test, $p< 0.001$ (***). Also see Source Data 4.

Biology Open • Supplementary information

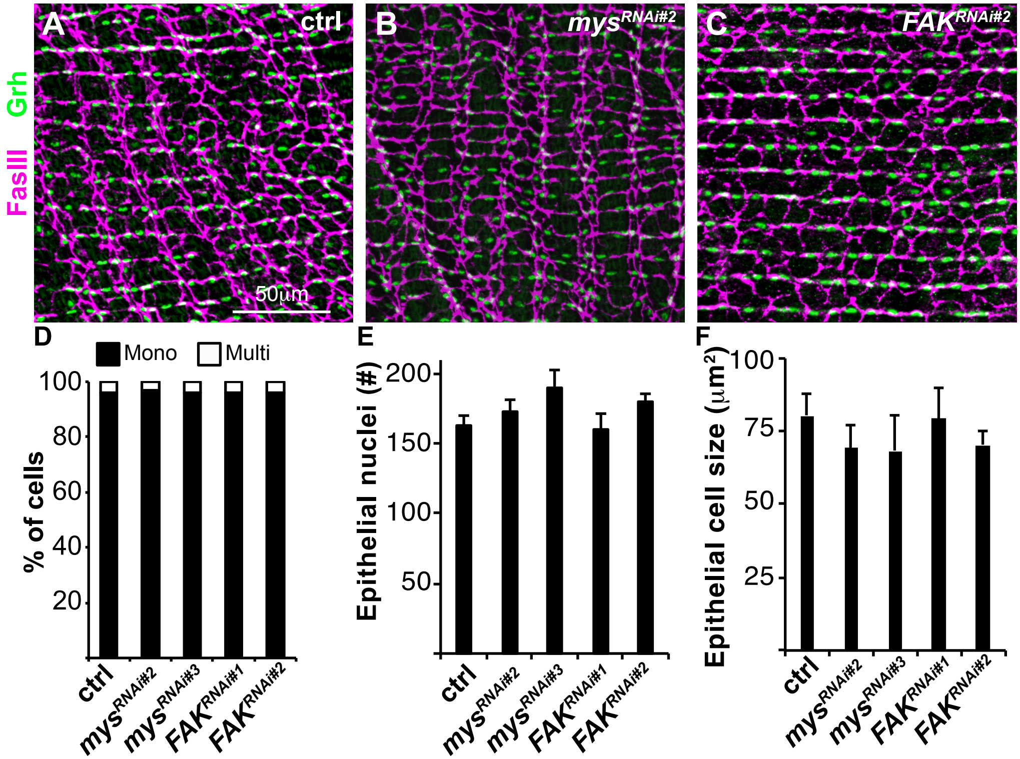

Figure S2. Focal adhesion gene RNAi does not cause ectopic cell fusion. (A-C) Representative immunofluorescent images of FasIII and Grh staining in the uninjured fly abdomen from control, *mys*^{RNAi}, and *Fak*^{RNAi}. (D-F) Quantification of the number nuclei per cell (D), total number of epithelial nuclei (E), and epithelial cell size (F) for control (n=9), *mys*$^{RNAi\#3}$(n=9), *Fak*$^{RNAi\#1}$(n=9), *Fak*$^{RNAi\#2}$ (n=8). There is no significant difference in the number of multinucleated cells, epithelial nuclei, or epithelial cell size. Error bars represent mean $\pm$ SE and data were analyzed by two-tailed unpaired t-test. Also see Source Data 5.

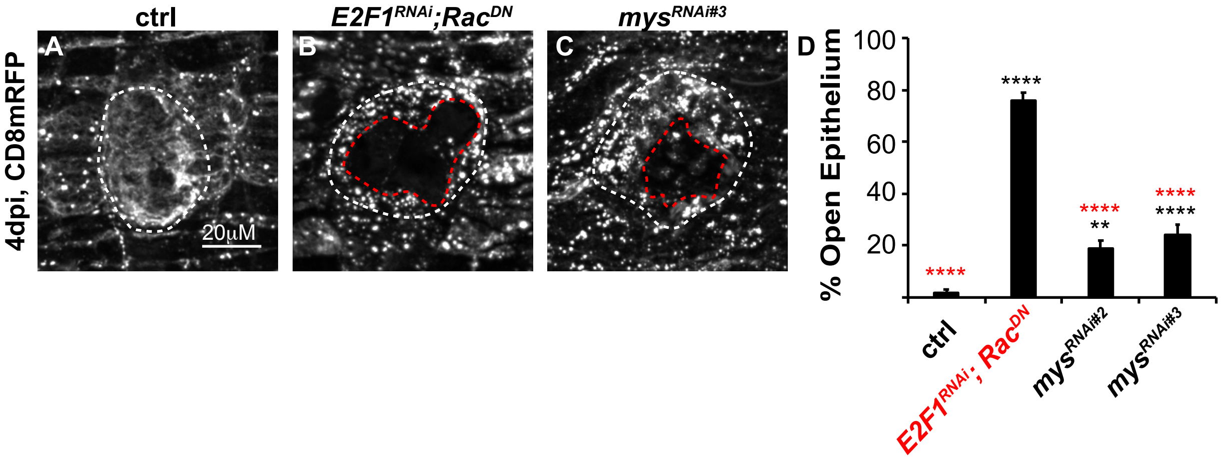

Figure S3. *Mys* knockdown delays wound closure. Re-epithelization during wound repair is detected by expression of a membrane-linked RFP (UAS-mCD8-RFP) under epi-Gal4 control. Representative immunofluorescent images for (A) control (epi-Gal4/+), (B) $E2F1^{RNAi}$; Rac^{DN}, and (C) $mys^{RNAi\#3}$ at 4 dpi. Outlined are wound scar (dashed white line) and open epithelial area (dashed red line). (C) Percent open area (open epithelial area/ wound scar size) at 4 dpi for control (n=21), $E2F1^{RNAi}$, Rac^{DN} (n=15), $mys^{RNAi\#2}$ (n=20), and $mys^{RNAi\#3}$ (n=20). Data were analyzed by 1-way ANOVA with Tukey's multiple comparisons test ** ($p<0.01$) and ****($p<0.0001$). Comparisons to control (black *) and $E2F1^{RNAi}$, Rac^{DN} (red *). Also see Source Data 6.
